# Supplementary material for: The role of traditional Chinese medicine on fracture surgery, hospitalization, and total mortality risks in diabetic patients with osteoporosis
Source: PLoS One. 2024 May 2;19(5):e0289455. doi: 10.1371/journal.pone.0289455 (PMC11065294; doi:10.1371/journal.pone.0289455)
Supplement: S1 Table — (DOCX) [file pone.0289455.s001.docx]

| **Supplemental table 1. Abbreviation, ICD-9-CM, and definition** | | |
| --- | --- | --- |
|  | **Abbreviation** | **ICD-9-CM / definition** |
| **Study population** |  |  |
| Diabetes mellitus | DM | 250, outpatient ≧ 3 visits or inpatient |
| Osteoporosis |  | 733.0, outpatient ≧ 3 visits or inpatient |
| **Intervention:** Traditional Chinese medicine | TCM |  |
| Herbal formulae |  |  |
| Supplemented free wanderer powder |  | Jia-Wei-Xiao-Yao-San |
| Channel-coursing blood-quickening decoction |  | Shu-Jing-Huo-Xue-Tang |
| Spiny jujube decoction |  | Suan-Zao-Ren-Tang |
| Sweet dew beverage |  | Gan-Lu-Yin |
| Stomach-calming powder |  | Ping-Wei-San |
| Pueraria decoction |  | Ge-Gen-Tang |
| Costusrootand amomum six gentlemen decoction |  | Xiang-Sha-Liu-Jun-Zi-Tang |
| Loniceraand forsythia powder |  | Yin-Qiao-San |
| Minor bupleurum decoction |  | Xiao-Chai-Hu-Tang |
| Pinellia heart draining decoction |  | Ban-Xia-Xie-Xin-Tang |
| Acupuncture |  |  |
| TCM traumatology |  |  |
| **Events:** Prognosis |  |  |
| Fracture surgery |  | 733.1, 800 - 829 & Surgery cost > 0 |
| Inpatient |  |  |
| All-caused mortality |  | 001 - 999, E800 - E999 |
| **Charlson comorbidity index revised** | CCI_R | CCI removed DM |
