# Supplementary material for: The role of traditional Chinese medicine on fracture surgery, hospitalization, and total mortality risks in diabetic patients with osteoporosis
Source: PLoS One. 2024 May 2;19(5):e0289455. doi: 10.1371/journal.pone.0289455 (PMC11065294; doi:10.1371/journal.pone.0289455)
Supplement: S2 Table — (DOCX) [file pone.0289455.s002.docx]

| **Supplemental table 2 factors of fracture, inpatient, all-caused mortality stratified by variables listed in the table by using Cox regression** | | | | | | |
| --- | --- | --- | --- | --- | --- | --- |
| **TCM** | **Fracture** | | | | | |
|  | **With** | | | **Without** *(Reference)* | | |
| **Stratified** | **Events** | **PYs** | **Rate (per 10^5^ PYs)** | **Events** | **PYs** | **Rate (per 10^5^ PYs)** |
| **Total** | 433 | 30,014.22 | 1,442.65 | 617 | 30,137.92 | 2,047.25 |
| **Gender** |  |  |  |  |  |  |
| Male | 144 | 9,825.97 | 1,465.50 | 203 | 9,866.27 | 2,057.52 |
| Female | 289 | 20,188.25 | 1,431.53 | 414 | 20,271.65 | 2,042.26 |
| **Age groups (yrs)** |  |  |  |  |  |  |
| 18 - 49 | 33 | 2,588.27 | 1,274.98 | 53 | 2,597.24 | 2,040.63 |
| 50 - 49 | 82 | 6,116.90 | 1,340.55 | 124 | 6,073.42 | 2,041.68 |
| ≧ 60 | 318 | 21,309.05 | 1,492.32 | 440 | 21,467.26 | 2,049.63 |
| **Catastrophic illness** |  |  |  |  |  |  |
| Without | 336 | 23,600.92 | 1,423.67 | 503 | 24,622.68 | 2,042.83 |
| With | 97 | 6,413.30 | 1,512.48 | 114 | 5,515.24 | 2,067.00 |
| **TCM** | **Inpatient** | | | | | |
|  | **With** | | | **Without** *(Reference)* | | |
| **Stratified** | **Events** | **PYs** | **Rate (per 10^5^ PYs)** | **Events** | **PYs** | **Rate (per 10^5^ PYs)** |
| **Total** | 624 | 32,597.21 | 1,914.27 | 835 | 32,603.11 | 2,561.11 |
| **Gender** |  |  |  |  |  |  |
| Male | 211 | 10,671.52 | 1,977.23 | 274 | 10,673.34 | 2,567.14 |
| Female | 413 | 21,925.69 | 1,883.64 | 561 | 21,929.77 | 2,558.17 |
| **Age groups (yrs)** |  |  |  |  |  |  |
| 18 - 49 | 50 | 2,811.06 | 1,778.69 | 71 | 2,790.69 | 2,544.17 |
| 50 - 49 | 124 | 6,643.35 | 1,866.53 | 168 | 6,571.12 | 2,556.64 |
| ≧ 60 | 450 | 23,142.80 | 1,944.45 | 596 | 23,241.30 | 2,564.40 |
| **Catastrophic illness** |  |  |  |  |  |  |
| Without | 484 | 25,632.08 | 1,888.26 | 681 | 26,636.86 | 2,556.61 |
| With | 140 | 6,965.13 | 2,010.01 | 154 | 5,966.25 | 2,581.19 |
| **TCM** | **All-cause mortality** | | | | | |
|  | **With** | | | **Without** *(Reference)* | | |
| **Stratified** | **Events** | **PYs** | **Rate (per 105 PYs)** | **Events** | **PYs** | **Rate (per 10^5^ PYs)** |
| **Total** | 318 | 45,131.27 | 704.61 | 390 | 47,656.07 | 818.36 |
| **Gender** |  |  |  |  |  |  |
| Male | 107 | 14,774.65 | 724.21 | 129 | 15,605.24 | 826.65 |
| Female | 211 | 30,356.62 | 695.07 | 261 | 32,050.83 | 814.33 |
| **Age groups (yrs)** |  |  |  |  |  |  |
| 18 - 49 | 28 | 3,891.75 | 719.47 | 35 | 4,077.15 | 858.44 |
| 50 - 49 | 65 | 9,096.23 | 714.58 | 80 | 9,608.23 | 832.62 |
| ≧ 60 | 225 | 32,143.29 | 699.99 | 275 | 33,970.69 | 809.52 |
| **Catastrophic illness** |  |  |  |  |  |  |
| Without | 247 | 35,488.09 | 696.01 | 318 | 38,934.63 | 816.75 |
| With | 71 | 9,643.18 | 736.27 | 72 | 8,721.44 | 825.55 |
| PYs = Person-years; Adjusted HR = Adjusted Hazard ratio: Adjusted for the variables listed in Table 3.; CI = confidence interval | | | | | | |
