# Supplementary material for: The role of traditional Chinese medicine on fracture surgery, hospitalization, and total mortality risks in diabetic patients with osteoporosis
Source: PLoS One. 2024 May 2;19(5):e0289455. doi: 10.1371/journal.pone.0289455 (PMC11065294; doi:10.1371/journal.pone.0289455)
Supplement: S3 Table — 1. Years of follow-up. 2. Years to prognosis. (DOCX) [file pone.0289455.s003.docx]

| **Supplemental table 3-1. Years of follow-up** | | | | | |
| --- | --- | --- | --- | --- | --- |
| **TCM** | **Min** | **Median** | **Max** | **Mean ± SD** | ***P*** |
| With | 0.36 | 7.98 | 15.90 | 10.61 ± 7.92 |  |
| Without | 0.40 | 8.42 | 15.92 | 10.90 ± 8.14 |  |
| Total | 0.36 | 8.23 | 15.92 | 10.76 ± 8.03 | 0.153 |
| ***P*: t-test** | | | | | |

| **Supplemental table 3-2. Years to prognosis** | | | | | | | | | | | | | | | |
| --- | --- | --- | --- | --- | --- | --- | --- | --- | --- | --- | --- | --- | --- | --- | --- |
| **Prognosis** | **Fracture surgery** | | | | | **Inpatient** | | | | | **All-caused mortality** | | | | |
| **TCM** | **Min** | **Median** | **Max** | **Mean ± SD** | ***P*** | **Min** | **Median** | **Max** | **Mean ± SD** | ***P*** | **Min** | **Median** | **Max** | **Mean ± SD** | ***P*** |
| With | 0.42 | 4.03 | 15.24 | 4.91 ± 3.76 |  | 0.48 | 4.29 | 15.64 | 5.29 ± 4.48 |  | 0.36 | 5.30 | 15.90 | 6.61 ± 6.03 |  |
| Without | 0.49 | 4.18 | 15.41 | 5.18 ± 3.92 |  | 0.53 | 4.47 | 15.78 | 5.77 ± 4.79 |  | 0.40 | 5.76 | 15.90 | 7.04 ± 6.22 |  |
| Total | 0.42 | 4.10 | 15.33 | 5.05 ± 3.84 | 0.005 | 0.48 | 4.38 | 15.71 | 5.53 ± 4.64 | < 0.001 | 0.36 | 5.44 | 15.90 | 6.32 ± 6.65 | 0.001 |
| ***P*: t-test** | | | | | | | | | | | | | | | |
