# Supplementary material for: The role of traditional Chinese medicine on fracture surgery, hospitalization, and total mortality risks in diabetic patients with osteoporosis
Source: PLoS One. 2024 May 2;19(5):e0289455. doi: 10.1371/journal.pone.0289455 (PMC11065294; doi:10.1371/journal.pone.0289455)
Supplement: Attachment — Submitted filename: Response to reviewer - PONE-D-23-22181 R1.doc [file pone.0289455.s004.doc]

**The role of traditional Chinese medicine on fracture surgery, hospitalization, and total mortality risks in diabetic patients with osteoporosis**

**RE:PLOS ONE Decision: Revision required [PONE-D-23-22181]**

**Lu et al.**

**Reviewer #1**: The causes of fracture are multiple factorial. Thus, the TCM treatment reduce fracture rate may through other route. For example, improve eye function thus reduce falling events, thus reduce fracture rate. I hope the authors can have more works about this.

Response:

Thank you for your suggestions.

We describe in the discussion a study showing the efficacy of traditional Chinese medicine in the treatment of age-related macular degeneration by improving the vision of diabetic patients with retinal macular degeneration in Page 17 Line 276-283.

Thank you again for pointing these out.

Thank you for your encouragement and I will continue to work hard to write relevant articles.

**Reviewer #2**: This study utilized a nationwide health database to analyze the use of traditional Chinese medicine(TCM) among diabetic patients with or without osteoporosis and its impact on health outcomes. This is a highly innovative research approach. I have the following major comments for the authors:

1. In the Materials and Methods section, the author did not provide clear definitions for diabetes, osteoporosis, and health outcomes, especially considering that a claims database was used as the data source.

Response:

Thank you for your suggestions.

We have elaborated and mentioned the definition for diabetes, osteoporosis, and add supplementary table 1 to illustrate in Page 7 Line 118-121. “National Health Insurance Research Database uses the International Classification of Diseases, Ninth Revision Clinical Modification (ICD-9-CM) to record diagnoses. All diagnoses of type 2 diabetes mellitus and osteoporosis are made by specialist certified medical professionals.”

Thank you again for pointing these out.

2. There is a lack of specific drug classification explanation for the use of TCM scientifically processed (Powdered)

Response:

Thank you for your suggestions.

We have elaborated and added on Table 5 (factors of prognosis among different TCM subgroups by using Cox regression) and supplementary table 1 (distribution of herbal formulae) to describe the information of TCM usage in this population including their types of treatment such as

1. Herbal formulae only
2. Acupuncture only
3. TCM traumatology only
4. Herbal formulae + Acupuncture
5. Herbal formulae + TCM traumatology

In Page 14-15 Line 245-252.

Thank you again for pointing these out.

3. The definition of censoring is not explicitly explained.

Response:

Thank you for your suggestions.

We have added the following description to the Materials and Methods section as "We included 29,166 T2DOP, and excluded 5,136 patients who received TCM treatment before 2000, those who had no follow-up records, those who were younger than 18 years old, and whose gender could not be identified, finally included 24030 T2DOP. Among them, there were 4155 patients who received TCM treatment, 19875 patients who did not receive TCM treatment, and 1024 outpatient follow-ups who received TCM treatment were excluded for less than 3 times. The remaining 3131 patients who received TCM treatment were divided by 1-fold propensity score matching by gender, age, and inclusion date as control group without TCM treatment as shown in Figure 1” in Page 8 Line 133-141.

Thank you again for pointing these out.

4. For research results not provided Incidence rates for the main outcomes.

Response:

Thank you for your suggestions.

We have elaborated and added on supplementary table 2 to describe the factors of fracture, inpatient, all-caused mortality stratified by variables listed in the table by using Cox regression in the Page 14 Line 240-243.

Thank you again for pointing these out.

5. There is no discussion on the selection bias and its possible impact on research results.

Response:

Thank you for your suggestions.

When seeing a Chinese medicine doctor in Taiwan, if the Chinese medicine practitioner prescribes water-cooked medicine, the health insurance will not pay for the medicine. Most medical institutions will let the patient pay for the treatment at his own expense. As a result, there is no information in the health insurance database, so the results may be underestimated.

We have elaborated and describe the limitation in Page 20 Line 340-344.

Thank you again for pointing these out.

6. Please ensure that references cited in the text are consistent.

Response:

Thank you for your suggestions.

We have re-checked the arrangement of the reference using endnote software, and it has been confirmed to be correct.

Thank you again for pointing these out.

**Reviewer #3:** PONE-D-23-22181: statistical review

SUMMARY.

This is a retrospective study to test whether traditional Chinese therapy reduces the risk of fracture surgery, hospitalization and total mortality in a sample of diabetic patients with osteoporosis. The core statistical analysis relies on a battery of Cox regressions models, estimated from two propensity-score matched samples. Beside the problematic intelligibility of the paper (see specific point no. 1), there are some major issues that should be addressed.

MAJOR ISSUES

1. Propensity-score matched samples can be useful to alleviate the possible bias of retrospective studies like this one. However, the matching is successful when there are not significant differences between the groups of interest (in this case the groups with and without TCM treatments). This is unfortunately not the case in this study (see table 1) because there are relevant differences in level of care and catastrophic illness. The authors should discuss why these differences are not expected to bias the results.

Response:

Thank you for your suggestions.

1. We only match basic demographic characteristics and inclusion time, without matching level of care and catastrophic illness, to avoid over-matching and obscuring an exist risk.
2. We based on the article “Negative and non-positive epidemiological studies. Olav Axelson. Int J Occup Med Environ Health. 2004;17(1):115-21.” as this study designed by over-matching.

Thank you again for pointing these out.

2. The treatment of interest is traditional Chinese medicine, but nothing is said about this treatment. Is there a specific TCM treatment for diabetic patients with osteoporosis? How long have these patients been treated by TCM?

Response:

Thank you for your suggestions.

We have elaborated and added on Table 5 (factors of prognosis among different TCM subgroups by using Cox regression) and supplementary table 1 (distribution of herbal formulae) to describe the information of TCM usage in this population including their types of treatment such as

1. Herbal formulae only
2. Acupuncture only
3. TCM traumatology only
4. Herbal formulae + Acupuncture
5. Herbal formulae + TCM traumatology

In Page 14-15 Line 245-252.

We have elaborated and added on supplementary table 3 to mention the average follow-up time and other information in Page 14 Line 243.

Thank you again for pointing these out.

3. The study relies on very few covariates and quite high is the risk that unobserved confounding factors could bias the analysis. I assume that the authors did not include further covariates simply because they are not available in the dataset. Undesired effects of unobserved confounders could be alleviated by introducing random effects in the Cox regression model, a method that is available in SPSS, the software used by the authors.

Response:

Thank you for your suggestions.

We have elaborated and revised our Statistical Analysis as : “ All statistical analyzes were performed using SPSS software version 22 (SPSS Inc., Chicago, IL, USA), R software version 4.3.1 (R Software Inc., San Francisco, CA, USA) and STATA software vesion 9 (StataCorp LLC., College Station, Texas, USA). Chi-square and t-tests were used to assess the distribution of categorical and continuous variables, respectively. Multivariate Cox proportional hazards regression analysis with mixed effects model was used to determine the risk of fracture surgery, hospitalization, or death in T2DOP treated with TCM. We used Schoenfeld's global test to test the proportional hazards assumption in the Cox proportional hazards model. Results of statistical analyzes are presented as hazard ratios (HR) and 95% confidence intervals (CI). Differences in the risk of fracture surgery, hospitalization, or death between groups receiving and not receiving TCM were estimated using the Kaplan-Meier method and the log-rank test. Statistical significance was determined using a two-tailed test with a p-value less than 0.05.” in Page 9 Line 150-158.

Thank you again for pointing these out.

SPECIFIC POINTS.

1. English. The paper has several grammar and language issues, which need to be addressed. Even the title does not seem grammatically correct to me. A title like "The role of traditional Chinese medicine on fracture surgery, hospitalization and total mortality risks in diabetic patients with osteoporosis" would sound better.

Response:

Thank you for your suggestions.

We have changed the title to "The role of traditional Chinese medicine on fracture surgery, hospitalization and total mortality risks in diabetic patients with osteoporosis" as you suggested. We have also sent the article for English revision and attached a revision certificate. (GwoJang Tech Inc. Medical Editing & Review Certificate. TEL： +886 2 23514696; FAX：+886 2 23514696; kc2351@ms24.hinet.net)

Thank you again for pointing these out.

2. The Cox regression model relies on the hypothesis of proportional hazards. Could the authors provide evidence that such assumption is fulfilled by the data?

Response:

Thank you for your suggestions.

We have elaborated and revised our Statistical Analysis as : “ All statistical analyzes were performed using SPSS software version 22 (SPSS Inc., Chicago, IL, USA), R software version 4.3.1 (R Software Inc., San Francisco, CA, USA) and STATA software vesion 9 (StataCorp LLC., College Station, Texas, USA). Chi-square and t-tests were used to assess the distribution of categorical and continuous variables, respectively. Multivariate Cox proportional hazards regression analysis with mixed effects model was used to determine the risk of fracture surgery, hospitalization, or death in T2DOP treated with TCM. We used Schoenfeld's global test to test the proportional hazards assumption in the Cox proportional hazards model. Results of statistical analyzes are presented as hazard ratios (HR) and 95% confidence intervals (CI). Differences in the risk of fracture surgery, hospitalization, or death between groups receiving and not receiving TCM were estimated using the Kaplan-Meier method and the log-rank test. Statistical significance was determined using a two-tailed test with a p-value less than 0.05.” in Page 9 Line 150-158.

The P value of the Global test is not significant (P > 0.05), which means that the assumption of Cox regression is not violated.

We have elaborated and add on a note below the submitted Table 3: Global test, P = 0.782 (Fracture surgery), 0.645 (Inpatient), and 0.893 ( All-caused mortality)

Thank you again for pointing these out.

**Reviewer #4**: The manuscript entitled “Association with Lowering Fracture, Inpatient and Mortality Risk in Patients of Diabetes with Osteoporosis Combined Traditional Chinese Medicine Therapy” is presented. The study is potentially interesting. The methods employed for analyses were reasonable and the conclusions were justified. There are some potential issues.

1. Tabels 1 and table 4, Do you have Dexa scores for Patients of Diabetes? It was mentioned that TCM has effects on osteoporosis. Recent studies have found Therapeutic Potential and Outlook of Alternative Medicine in bone conditions via pro-anabolic effects and anti-catabolic effects and the regulation of osteoclasts and osteoblasts (for example PMID: 28325144, PMID: 31824310). It would be relevant to discuss the osteoblastic effects and osteoclastic effects of TCM and how this might affect the precited association with Lowering Fracture, Inpatient and Mortality Risk in Patients of Diabetes with Osteoporosis.

Response:

Thank you for your suggestions.

This study uses the health insurance database for analysis. There is no Dexa scores for patients with diabetes and we also write it into the limitation in Page 20 Line 338-340.

We have elaborated the important and relevant research findings to be conveyed in these two articles provided by the reviewer in Page 18 Line 307-309, as reference 29 and 33.

Thank you very much for providing relevant literature ”Therapeutic Potential and Outlook of Alternative Medicine for Osteoporosis”; “Therapeutic Anabolic and Anticatabolic Benefits of Natural Chinese Medicines for the Treatment of Osteoporosis” to make the content of this article more complete.

Thank you again for pointing these out.

2. Can you include serum biomarkers of bone remodelling in these analyses? such as measurement data of b-CTX,TRAP-5b , P1NP, ALP? In this study, it was mentioned that TCM has multiple effects. Many studies have found (for example PMID: 22465238) bone homeostasis is regulated by osteoclasts and osteoblasts together with osteocytes, bone lining cells, osteomacs, and vascular endothelial cells in the bone microenvironment within the basic multicellular unit (BMU). It would be informative to discuss the effect of diabetes on these cell types.

Response:

Thank you for your suggestions.

This study uses the health insurance database for analysis. There is no b-CTX, TRAP-5b, P1NP, or ALP and we write it into the discussion and limitation in Page 20 Line 338-340.

We have elaborated the important and relevant research findings to be conveyed in this article provided by the reviewer in Page 18-19 Line 309-312, as reference 34.

Thank you very much for providing relevant literature as “An overview of the regulation of bone remodelling at the cellular level” to make the content of this article more complete.

Thank you again for pointing these out.
