# Supplementary material for: The role of traditional Chinese medicine on fracture surgery, hospitalization, and total mortality risks in diabetic patients with osteoporosis
Source: PLoS One. 2024 May 2;19(5):e0289455. doi: 10.1371/journal.pone.0289455 (PMC11065294; doi:10.1371/journal.pone.0289455)
Supplement: Attachment — Submitted filename: Response to reviewer PONE-D-23-22181.doc [file pone.0289455.s005.doc]

**Association with Lowering Fracture, Inpatient and Mortality Risk in Patients of Diabetes with Osteoporosis Combined Traditional Chinese Medicine Therapy**

**RE:PLOS ONE Decision: Revision required [PONE-D-23-22181-R1]**

**Lu et al.**

**Reviewer #2**: Thank you for the author's response to the review comments. The author did not conduct an analysis regarding the correlation between severity of diabetes, osteoporosis, and the cumulative amount of traditional Chinese medicine usage. It is suggested that the author discuss the impact of diabetes, osteoporosis severity and indication bias on the study results to enhance this better of this manuscript.

Response:

Thank you for your suggestions.

We describe in the discussion with blue words in Line 344-351, Page 20-21.

Thank you again for pointing these out.

**Reviewer #3**: PONE-D-23-22181R1: statistical review

SUMMARY. In my first review of this paper, I have raised 3 major issues and 2 specific points. Major issues no. 1 and 2 and specific issue no. 1 have been adequately addressed. However, there is problem with the remaining issues. See below.

1. I previously raised the following concern: "The study relies on very few covariates and quite high is the risk that unobserved confounding factors could bias the analysis. I assume that the authors did not include further covariates simply because they are not available in the dataset. Undesired effects of unobserved confounders could be alleviated by introducing random effects in the Cox regression model, a method that is available in SPSS, the software used by the authors." In the revised manuscript, the authors declare that the statistical analysis has been revised by including random effects in the model. However, the results in Table 3 and 4 are the same as in the previous version. Table 5 seems also to have been done without including random effects. I would like to see the new tables that have been obtained by a mixed effect Cox regression.

Response:

Thank you for your suggestions.

We are sorry that the data was not updated last time. We have corrected it, as shown in red letters in the attached Table.

The parts where the correction content is modified in abstracts and results are also displayed in blue.

Thank you again for pointing these out.

2. I previously raised some concerns about the English. Although the authors declare that the English has been revised, I still see mistakes.

Response:

Thank you for your suggestions.

The files for tracking revisions include corrections related to English revisions in the article.

Thank you again for pointing these out.

**Reviewer #4**: This is a revised paper. The authors have addressed questions and the paper has been improved. It is acceptable for publication.

Response:

Thank you for your previous advice.

Thank you for the very kind encouragement
