# Supplementary material for: The role of traditional Chinese medicine on fracture surgery, hospitalization, and total mortality risks in diabetic patients with osteoporosis
Source: PLoS One. 2024 May 2;19(5):e0289455. doi: 10.1371/journal.pone.0289455 (PMC11065294; doi:10.1371/journal.pone.0289455)
Supplement: Attachment — Submitted filename: Response to reviewer PONE-D-23-22181 R2.doc [file pone.0289455.s006.doc]

**Association with Lowering Fracture, Inpatient and Mortality Risk in Patients of Diabetes with Osteoporosis Combined Traditional Chinese Medicine Therapy**

**RE:PLOS ONE Decision: Revision required [PONE-D-23-22181-R2]**

**Lu et al.**

1. The distribution between the experimental and control groups differs, notably in the variable "catastrophic illness." Such difference may significantly impact outcomes, urging consideration of this variable during matching procedures.

Response:

Thank you for your suggestions.

Following the reviewer's suggestions, we make the distributions similar between the experimental and control groups at the beginning of the analysis and displayed the faithful description based on the new figures and tables in green fonts in the article.

Thank you again for pointing these out.

2. It is recommended to provide documentation verifying the edits made to the manuscript after revision. Some English errors persist, including for example, inaccurate descriptions in tables such as "hospital center.”

Response:

Thank you for your suggestions.

We have corrected it, as shown in green fonts in the attached Table.

Thank you again for pointing these out.

3. Inconsistencies exist regarding the terms "comorbidity" and "severity" throughout the article. Confirm that the Charlson Comorbidity Index (CCI) represents comorbidity, while catastrophic illness denotes severity. Including literature to support the revised CCI. There were many studies utilizing database-derived measure like the Diabetes Complications Severity Index (DCSI) to demonstrate severity for patients with diabetes. Please amend the "Limitations" section to reflect this aspect accurately.

Response:

Thank you for your suggestions.

1. We have corrected it, as shown in green fonts in Line 215, 222 Page 13 and Line 230 Page 14.
2. The reason why we did not use Diabetes Complications Severity Index (DCSI) is because DCSI requires biochemical index values, but the health insurance database does not have them, and it has been written into the limitation in Line 340-343, Page 20 with reference 38.

Thank you again for pointing these out.

4. Please explain the results of Schoenfeld's global test and what the assumptions required for proportional hazard analysis the test satisfies.

Response:

Thank you for your suggestions.

As a result of Schoenfeld's global test, if P > 0.05, it means that the Proportional Hazard Assumption is not violated. In other words, the Cox proportional hazards model can be used. Cox proportional hazards model assumes that the hazards of the different strata formed by the levels of the covariates are proportional.

Reference as *J.Natn.Sci.Foundation Sri Lanka* 2009 37(1):41-5. “Use of Schoenfeld’s global test to test the proportional hazards assumption in the Cox proportional hazards model: an application to a clinical study”

Thank you again for pointing these out.
