# Supplementary material for: The role of traditional Chinese medicine on fracture surgery, hospitalization, and total mortality risks in diabetic patients with osteoporosis
Source: PLoS One. 2024 May 2;19(5):e0289455. doi: 10.1371/journal.pone.0289455 (PMC11065294; doi:10.1371/journal.pone.0289455)
Supplement: Attachment — Submitted filename: Response to reviewer PONE-D-23-22181 R3.doc [file pone.0289455.s007.doc]

**The role of traditional Chinese medicine on fracture surgery, hospitalization, and total mortality risks in diabetic patients with osteoporosis**

**RE:PLOS ONE Decision: Revision required [PONE-D-23-22181-R3]**

**Lu et al.**

**Journal Requirements:**

Please review your reference list to ensure that it is complete and correct. If you have cited papers that have been retracted, please include the rationale for doing so in the manuscript text, or remove these references and replace them with relevant current references. Any changes to the reference list should be mentioned in the rebuttal letter that accompanies your revised manuscript. If you need to cite a retracted article, indicate the article’s retracted status in the References list and also include a citation and full reference for the retraction notice.

Response:

Thank you for your suggestions.

We have followed the journal requirements to check all reference lists to ensure they are complete and correct.

**Additional Editor Comments:**

1. There may be more than one instance of grammatical issues (the reviewer just proposed an example). The editing certification is on November 4, 2023, and the manuscript has undergone several revisions after November 4, 2023. It is advisable to provide newest editing certification.

Response:

Thank you for your suggestions.

During the submission period, the manuscript has been revised after many reviewers' suggestions. We have also sent the last revised version to the English editing unit for revision, and provided the latest editorial certification. The date of completion of editing certification is Mar 14, 2024.

Thank you again for pointing these out.

2 Please include this description of Schoenfeld's global test and its underlying assumptions, inclusive of relevant literature, in the manuscript.

Response:

Thank you for your suggestions.

We have mentioned it, as shown in orange color words in Line 156-160 Page 9 in Statistical Analysis of Materials and Methods section.

Cox proportional hazards model assumes that the hazards of the different strata formed by the levels of the covariates are proportional. Schoenfeld's global test is used to test the residuals of Cox proportional model. If P > 0.05, it means that the proportional hazard assumption is not violated; in other words, the Cox proportional hazards model can be used.

With references 13 and 14.

13.	SCHOENFELD D. Chi-squared goodness-of-fit tests for the proportional hazards regression model. Biometrika. 1980;67(1):145-53. doi: 10.1093/biomet/67.1.145.

14.	Abeysekera WWM, Sooriyarachchi MR. Use of Schoenfeld’s global test to test the proportional hazards assumption in the Cox proportional hazards model: an application to a clinical study. Journal of the National Science Foundation of Sri Lanka. 2009. doi: 10.4038/jnsfsr.v37i1.456.

Thank you again for pointing these out.
